# Covid-19 vaccination: a mixed methods analysis of health system resilience in Latin America

**DOI:** 10.1186/s12939-023-02073-4

**Published:** 2024-02-12

**Authors:** Estefania Hernandez-Pineda, Ciro-Alberto Amaya, Catalina González-Uribe, Andrea Herrera, Nubia Velasco

**Affiliations:** 1 School of Management, Universidad de Los Andes, Calle 21 # 1-20, Bogotá, 111711 Colombia; 2 School of Engineering, Universidad de Los Andes, Cra 1 E ste N° 19A – 40, Bogotá, 111711 Colombia; 3 School of Medicine, Universidad de Los Andes, Carrera 1 No 18 A - 10 42 Bloque Q Piso 8, Bogotá, 111711 Colombia

**Keywords:** covid-19 vaccination, Mix-methods research, Health system resilience, Health system shocks, Vaccine supply-scheme

## Abstract

**Background:**

This paper discusses the stages of health system resilience (HSR) and the factors underlying differences in HSR during the covid-19 pandemic, especially the vaccination challenge. We studied the relationship between vaccination strategies and macro-context backgrounds in 21 Latin American countries. Our objective was to capture the impact of those aspects in the SR and identify potential improvements for future crises and for vaccination programs in general.

**Methods:**

The study uses mixed-methods research to provide insights into how the countries’ backgrounds and vaccination strategies impact the HSR. Particularly, we used explanatory sequential mixed methods, which entails a quantitative-qualitative two-phase sequence. The quantitative phase was conducted using cluster and variance analysis, in which the HSR was measured using as a proxy the covid-19 vaccination outcomes in three cut-offs of reaching 25%, 50%, and 75% of population coverage. This approach allows us to discriminate covid-19 vaccination progress by stages and contrast it to the qualitative stage, in which we performed a country-case analysis of the background conditions and the changes in vaccination strategies that occurred during the corresponding dates.

**Results:**

The paper provides a rich comparative case analysis of countries, classifying them by early, prompt, and delayed performers. The results show that differences in vaccination performance are due to flexibility in adapting strategies, cooperation, and the ability to design multilevel solutions that consider the needs of various actors in the health ecosystem. These differences vary depending on the vaccination stage, which suggests the importance of acknowledging learning, diffusion, and feedback processes at the regional level.

**Conclusions:**

We identified the importance of societal well-being as an ideal country antecedent for high and sustained levels of performance in covid-19 vaccination. Whereas in other countries where the set-up and beginning phases were rough, the value of the operational decisions and the learning on the move regarding their own and their peers’ trajectories were crucial and were reflected in performance improvement. A contribution of this study is that the above-mentioned analysis was done using vaccination coverage cut-off points that allow a performance view that takes into consideration the stages of the vaccination progress and the learning process that goes with it. As well as framing this into the HSR shock cycles that allow to differentiate the stages of resilience on which countries must act.

## Background

The covid-19 vaccination was a universal challenge, coupled with global inequalities in research, development, and production capacity that resulted in diverse vaccination strategies across countries, with low- and middle-income countries often forced to import vaccines [1]. These inequalities persisted during negotiations due to financial limitations and poor participation of some developing countries. COVAX was created as an international mechanism to negotiate on behalf of the global south and ensure vaccine access [2].

Developing countries played a central role in determining covid-19 vaccination strategies, such as ensuring all population segments were reached [1]. In this role, Chile’s exemplary resilience in adapting and responding to the pandemic [3] raised questions about the assumption of homogeneous conditions in Latin America and sparked interest in examining the influence of each country’s macro-context background and vaccination strategies. This article examines the influence of the background on health system resilience (HSR) in Latin American countries. The background consists of the largely system-wide culture, economics, politics, and system characteristics [4] that can condition causal mechanisms to hinder or drive certain outcomes [5]. This background is represented by the “Sustainable Economic Development Assessment” (SEDA) index [6], and the HSR is measured through the proxy of vaccination performance indexes. The approach follows an explanatory sequential design [7] in two stages. The former uses quantitative analysis, including cluster and multivariate analysis, to answer the question: *Are there significant differences in the covid-19 vaccination outcomes among Latin American countries based on their SEDA?* The latter, considers the quantitative results, and examines how covid-19 vaccination strategies impact HSR through documentary analysis, allowing us to answer the question: *How do the covid-19 vaccination strategies affect the HSR?*

The article highlights varying HSR capacities in Latin America and their relevance for crisis management and cooperation. It also provides historical context and lessons from covid-19 vaccination efforts for improving the HSR and future planning in public health. The article is divided into five sections covering the concept of resilience, methodology, analysis, and results in the context of Latin American health systems, ending with a conclusion and discussion.

HSR particularly involves the abilities to prepare for, manage (i.e., adapting, sustaining, absorbing), and learn and transform from shocks [3, 8] through effective interaction of health system functions, including leadership and governance, information to the community, health workforce, financing, medical products, and service delivery [9]. The evidence of resilience and its potential for enhancement can be observed across the stages of a shock cycle: preparedness for shocks; shock on set and alert; shock impact and management; and recovery and learning [8].

Most studies focus on the shock impact and management, measuring HSR through capacities such as absorptive, adaptative, or transformative [10] and focus on a specific disturbance, primarily assessing health service delivery and health workforce issues [11]. However, a broader perspective that considers the influence of external factors such as economic and sociopolitical systems would be beneficial, fostering a context-driven approach to resilience [12]. A search of the Web of Science database was conducted using the query [“health system resilience” AND (“covid-19” OR covid)] in the category of ‘Topic’ including abstracts, titles, and keywords to identify previous research on HSR during the COVID-19 emergency. The search yielded 43 results, including four theoretical articles reviewing the discourse and conceptual usage around resilience theories [13] and 39 empirical studies. We classified these 39 articles into four categories according to the level of the unit of analysis that the articles study (i.e., individual, health system, or macro level). The resulting categories were: (i) service level maintenance; (ii) healthcare personnel characteristics; (iii) effectiveness of government decisions; and (iv) combinations of the previous three.

In the service level maintenance category, 22 out of 38 articles focused on measuring resilience as the health system’s capacity to maintain service levels during the pandemic (e.g., [14]); differences between the papers in this category are in terms of the specialty analyzed. In the healthcare personnel characteristics, 7 out of 39 papers focused on studying the capacities and characteristics of healthcare personnel helpful in a crisis, analyzing workforce attributes such as density [15], capacities [16], and experiences [17]; and also, the role of healthcare consumers in organizational design and technology usage [18, 19]. In the effectiveness of government decisions category, 7 out of 39 studies approach resilience by measuring the effectiveness of politics and government decisions during pandemics, economic challenges, antecedents, and future global challenges (e.g., [19]). In the last category, 2 out of 39 combinations of the previous three. For instance, Arsenault et al. [20] and Busse et al. [21] use a comprehensive analysis of the response to covid-19 considering the national economic and social environment, policies, and health system capacities. Both are comparative analyses, one considering countries from the global north and the other categorizing the countries regarding their income level.

A cross-cutting classification to the level of analysis is the comparative nature of the research, across the four categories presented above, 11 articles developed a cross-country analysis, where sampling was guided by interests in a particular region such as Africa (e.g., [22]); national characteristics such as systems with health insurance [23]; or contrasting characteristics in macro-economic terms [24]. The literature review reveals that Latin America has not been extensively studied in terms of its HSR. While Chile and Mexico have been characterized according to their income level in previous studies, there is still a need for a regional analysis that considers the unique aspects of each national context and operational decisions, and we aim to contribute to filling this gap.

In sum, the literature shows that the concept of resilience is appropriate for understanding crises such as covid-19, but most studies have focused too narrowly on a single level of analysis: the workforce, the health system, or the government capacities. We aim to fill this gap by analyzing both macro-context antecedents measured through the SEDA index, and the health system capacity measured through the covid-19 vaccination strategies, to understand the HSR in Latin American countries during the pandemic.

**Fig. 1:**
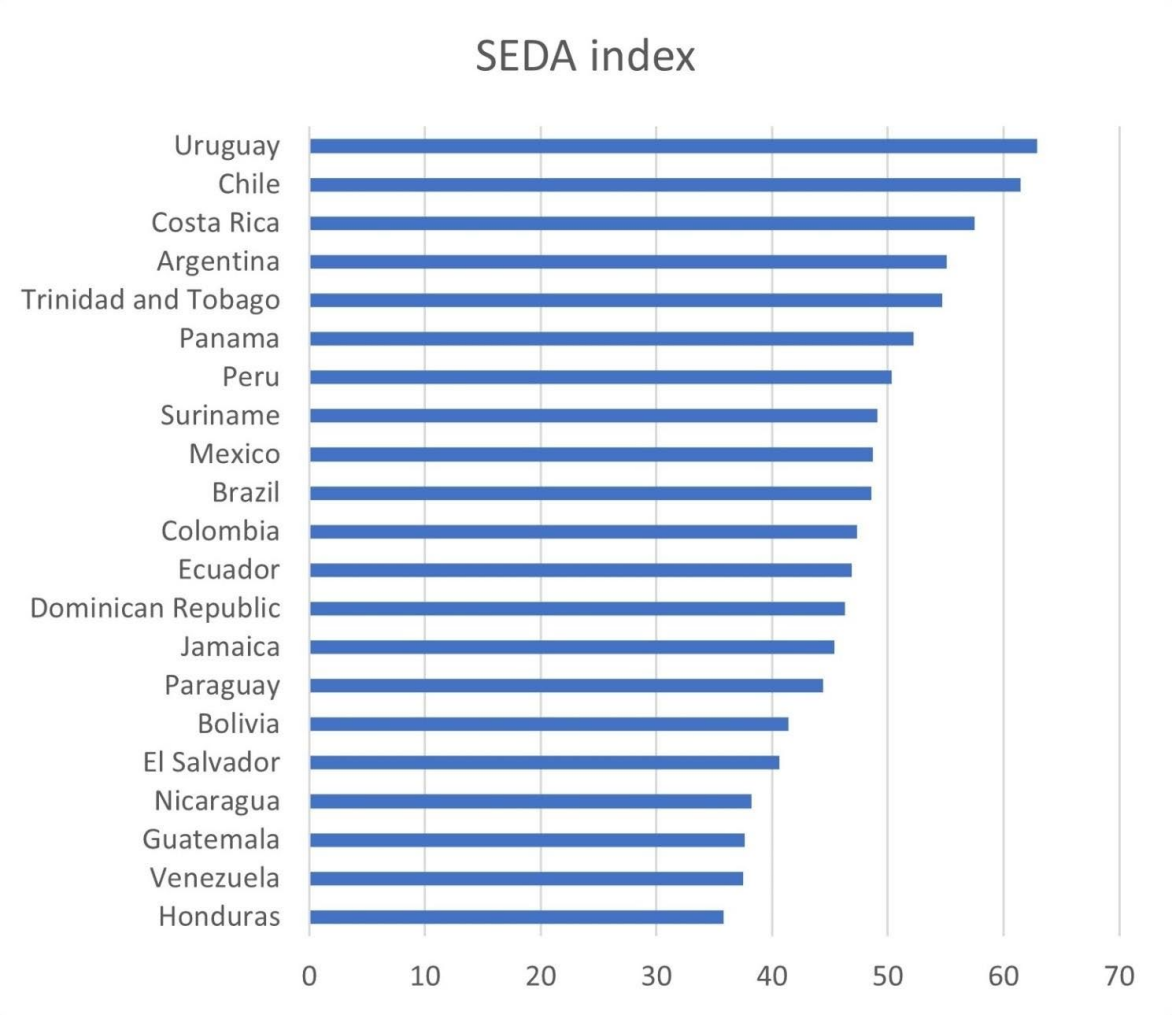
Latin American Countries with their respective SEDA score

Latin America comprises South America, Central America, Mexico, and some Caribbean islands resulting from a linguistic and cultural categorization of most Spanish-speaking countries that shared a colonial legacy, majoritarian Christianism beliefs, and common social and political institutions such as the civil law regime centralized governance [25]. Recognizing Latin America as a diverse and heterogeneous region is one of the main motivations for selecting it as the context of this research and filling the existent gap in the literature on cross-country analysis at the regional level. In addition to being Latin American countries, we considered the inclusion criteria of information available regarding each country. The study considers 21 countries, including Suriname, Trinidad and Tobago, and Jamaica from the Caribbean. Therefore, the list of Latin American countries considered in this study with their respective SEDA index is presented in Fig. 1.

Boston Consulting Group^1^ annually produces the SEDA index that provides an assessment of the macro-context background in terms of three categories: Economics, Investment, and Sustainability. These categories rely on some indicators that reveal the countries’ performance, and the indicators are produced upon the measurements taken in each country. We selected this index because it characterizes societal well-being across countries [26], which serves as a proxy for the macro-context background that interests us in this research. Table 1 presents the detail of the composition of the index. The values of each country in Fig. 1 correspond to their SEDA score for the year 2019, to consider the antecedent of countries before the covid-19 pandemic. For this study, we considered the overall SEDA index score, which takes values from 0 to 100, with 100 being the definition of an excellent macro-context background.

**Table 1:** SEDA index composition

Categories	Indicators	Measures
Economics	Income	Wealth
	Economic stability	Inflation; GDP and inflation volatility
	Employment	Rate of employment and unemployment
Investments	Health	Access to healthcare; healthcare outcomes
	Education	Access to education; education outcomes
	Infrastructure	Power; water; sanitation; transport; information and communications technology.
Sustainability	Environment	Quality of environment
	Governance	Effectiveness of government; accountability; stability; freedom.
	Civil society	Civic activism; intergroup cohesion; interpersonal safety and trust; gender equality
	Equality	Income distribution; equality in education and life expectancy.

As shown in Fig. 1, Chile and Uruguay have the two highest scores in the region, with the investment and sustainability categories contributing the most in these countries. In investment, Chile scored 74.8 and Uruguay 72.2, compared to the regional average of 57.4, while in sustainability Chile and Uruguay scored 56 and 62.6, respectively, while the average for the region is 46.1. If these categories are broken down, the main indicators contributing to these high scores in order of importance are infrastructure, health, and governance for Chile, and health, governance, and education for Uruguay. Conversely, Honduras and Venezuela obtained the lowest scores in the region, the two categories that brought their scores down were sustainability and economics, respectively. For Honduras, the main indicators hindering well-being are income, education, infrastructure, investment, and high inequality. The main indicators deteriorating the macro-context of Venezuela are economic stability, health investment, and governance.

## Methods

### Study design

**Fig. 2:**
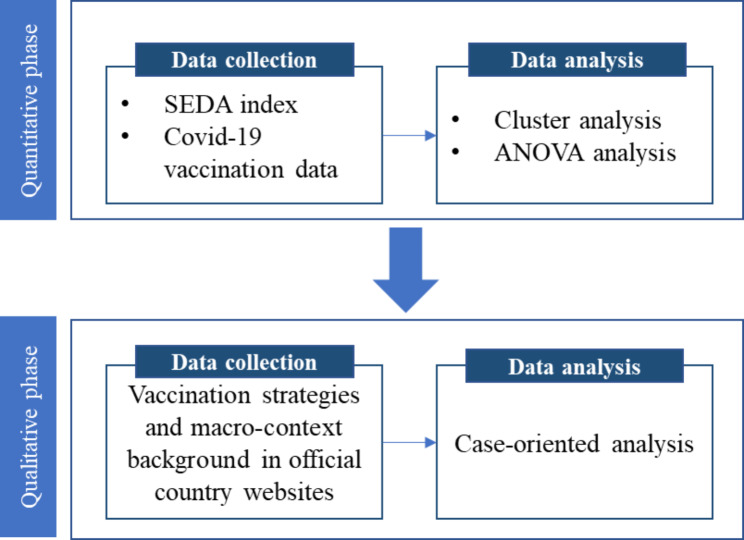
Detail of methodology: explanatory sequential design

The methodology selected to conduct this research is a mixed-methods approach. This approach aims to capture a comprehensive view of the health system and contextualize qualitative information gathered on country conditions by examining processes, experiences, and outcomes [27]. The design chosen to carry out the mixed methods approach was an explanatory sequential design [7] consisting of two consecutive phases (quantitative-qualitative) of data collection and analysis. Figure 2 presents the detail of the explanatory sequential design used in this study regarding data collection and data analysis procedures in each phase, which will be further explained in the remainder of this section.

### First phase: a quantitative approach

The purpose of this phase is to answer the question: *Are there significant differences in the covid-19 vaccination outcomes among Latin American countries based on their SEDA?* And pursues two main objectives: (1) to identify potential groups of the countries according to the SEDA index, which will be further explained in Sect. 4.1, and (2) to look for significant differences between them in terms of their performance in covid-19 vaccination, using analysis of variance. It is also expected to provide a general picture of the problem and preliminary relationships to guide the sampling and data collection of the next phase.

The methods selected for this quantitative approach were cluster analysis and analysis of variance (ANOVA). First, we developed a cluster analysis using complete linkage, since we needed to obtain country groups as different between them in terms of macro-context background, complete linkage was ideal because it groups observations by distancing the farthest neighbor [28]; in other words, forming groups by separating the countries according to how distinct they are from each other in terms of the SEDA index. The resulting classification serves as the independent variable in the analysis of variance. Second, the analysis of variance aims to identify whether there are significant differences among the groups of countries in terms of covid-19 vaccination outcomes. This analysis considers as a dependent variable the TTR of each threshold percentage of vaccinated inhabitants (25%-TTR25, 50%-TTR50, and 75%-TTR75). For both analyses, the statistical software Stata/SE version 17.0 was used, which enables users to analyze, manage, and produce graphical visualizations of the data [29]. We performed the analysis three times, one per each vaccinated population threshold, using the one-way command and the box-plot graphical analysis in Stata, the results are presented and discussed in Sect. 4.1.

The period of analysis of the vaccination outcomes spans from the start of covid-19 vaccination in each country until the end of January 2022. Thus, vaccination outcome is the dependent variable, and we measure it through the proportion of people vaccinated per country (i.e., the percentage of people vaccinated with at least one dose) taken from OWID database [30] and normalize it using the start date of each country to calculate the Time to Reach (TTR) 25%, 50%, and 75% respectively. The independent variable is the 2019 SEDA index.

Next, based on the findings from this quantitative stage, we sampled for the qualitative analysis. The second phase focuses on the qualitative analysis of decisions and strategies in the deployment of covid-19 vaccination that serve to understand differences in outcomes, from a preliminary review of the diverse strategies used globally.

### Second phase: a qualitative approach

The purpose of this phase is to deepen the previous results and refine the general picture [7] through a comparative case-oriented analysis, which is useful for ‘identifying common causal conditions linked to a specific outcome across a relatively small number of purposefully selected cases’ [31] (p.1141) In this study, the specific outcome that guided the case selection was the vaccination outcomes identified in the first phase of the study, in this phase the sampling criteria were: heterogeneity and diversity in terms of vaccination outcomes and macro-context background. Then, across the selected cases we looked out for causal conditions in the macro-context background and the vaccination strategies that could make understandable the vaccination outcomes which is the proxy for HSR. Thus, the second phase answers the question: *How do the covid-19 vaccination strategies affect the HSR?*

To answer that question, we identified three useful dimensions for this analysis: immunization coverage strategy (ICS), accessibility, and the covid-19 vaccine supply scheme of each country, which are detailed below. These dimensions were identified through a preliminary review of the vaccination strategies globally and were validated with experts’ opinions. These experts were part of the interdisciplinary group of scholars that formed COLEV, the project in which this study took place. The project had a committee of multidisciplinary experts, both national and international. We conducted validation meetings to receive feedback on the results of this article.

#### Immunization coverage strategy (ICS)

The review of the global vaccination process allowed us to identify two immunization approaches: prioritize at least one dose for most of the population or the completion of already initiated vaccination schemes. The ICS was measured by considering the proportion of fully vaccinated persons to the total number of doses administered per country. A low value on this ratio (close to zero) means that the government prioritized a high coverage of at least one dose in the majority of the population. On the contrary, a high ratio (close to 0.5) means that the country’s strategy was oriented towards completing schemes and guaranteeing the complete vaccination of people with schemes already initiated.

#### Accessibility

A zero-restriction model would be one in which individuals can be vaccinated by any service provider. This refers to the factors influencing the entry or use of health care services, in this case, covid-19 vaccination [32]. The information on restrictions was identified by analyzing the level of coordination in the roll-out in terms of consistency of all information sources found: patient requirements and compliance with the vaccination stages. In addition, efforts to facilitate access to vaccines for populations living in the peripheries were considered in the analysis as high accessibility.

#### Supply scheme

The supply scheme refers to how countries obtain vaccines; this involves the negotiation phase, participation in the COVAX mechanism, and the purchase and/or development of the vaccines. Also, the distribution channels are used to ensure the progress of the covid-19 vaccination plan in each country.

Information on these dimensions was gathered by searching secondary data on the official websites of each country. From these, we conducted a comparative case-oriented analysis by identifying the patterns of similarity and dissimilarity in the vaccination strategies across the selected cases and we built a categorization of cases that were representative of understanding the vaccination outcomes observed and that provide insights on its potential impact on HSR.

## Results

### First phase: quantitative results and analysis

**Fig. 3:**
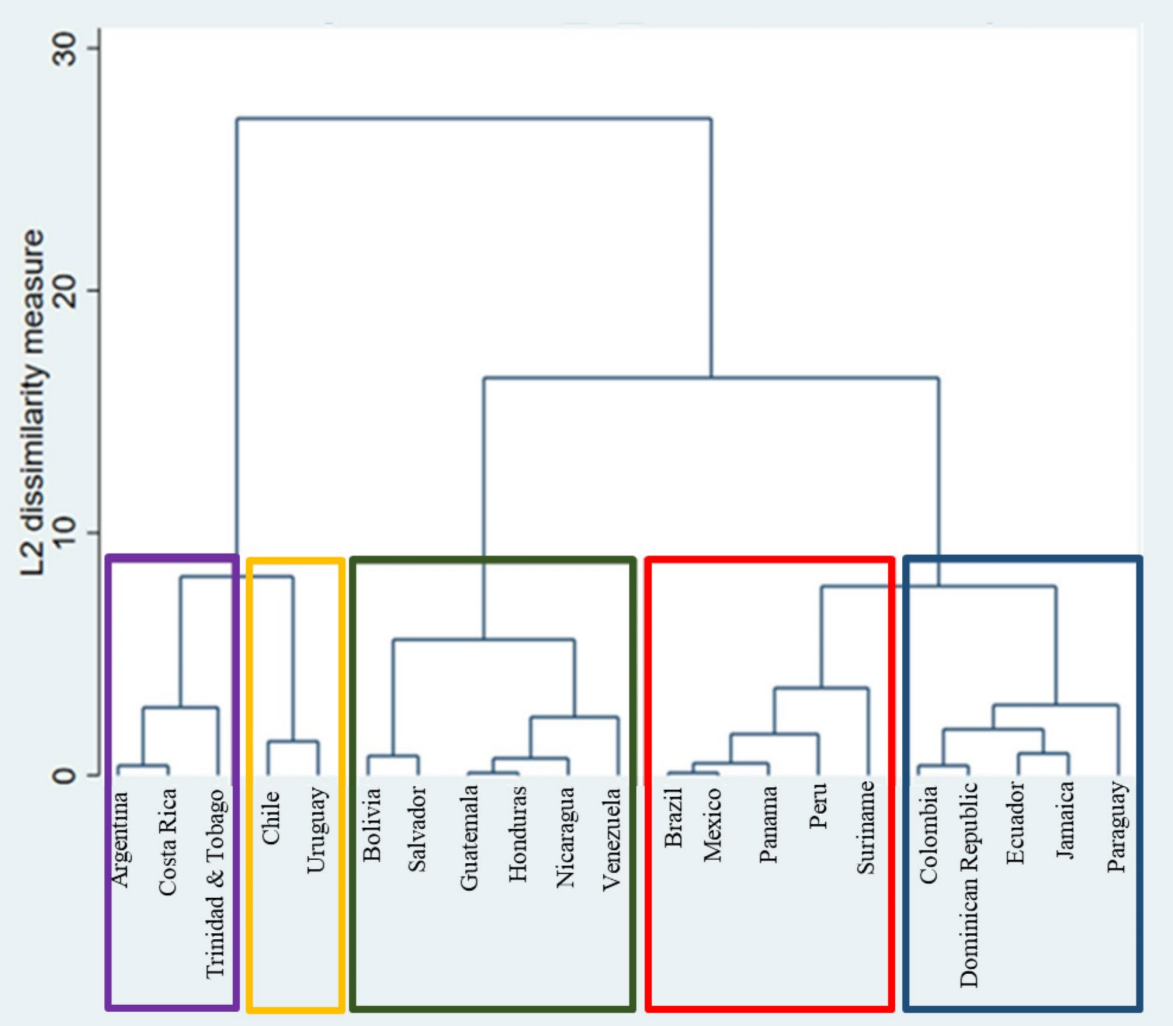
Dendrogram from complete linkage clustering of Latin American countries in SEDA index

The results of the clustering exercise grouped the countries according to their distinctiveness regarding the SEDA index. Five country groups emerged from the cluster analysis. Group 1 corresponds to Argentina, Costa Rica, and Trinidad & Tobago; Group 2 corresponds to Chile and Uruguay; Group 3 corresponds to Bolivia, El Salvador, Guatemala, Honduras, Nicaragua, and Venezuela; Group 4 corresponds to Brazil, Mexico, Panama, Peru, and Suriname; and group 5 corresponds to Colombia, Dominican Republic, Ecuador, Jamaica, and Paraguay. Figure 3 shows the dendrogram obtained after the cluster analysis. The five countries on the far left [1, 19, 6, 4, and 20] are the most dissimilar from the rest by approximately 10 points; these countries are Argentina, Trinidad & Tobago, Costa Rica, Chile, and Uruguay. However, within this group, internal differences are shown in the dendrogram at the right which shows that the appropriate grouping for those five countries is dividing them into three and two, corresponding to G1 (purple box) and G2 (yellow box), respectively. Next to the right, the countries from the green box in Fig. 3) constitute G3 and show a dissimilarity measure in the vertical axis lower than the other groups. While the other countries are equally divided into groups of five, comprising G4 (red box) and G5 (blue box).

**Fig. 4:**
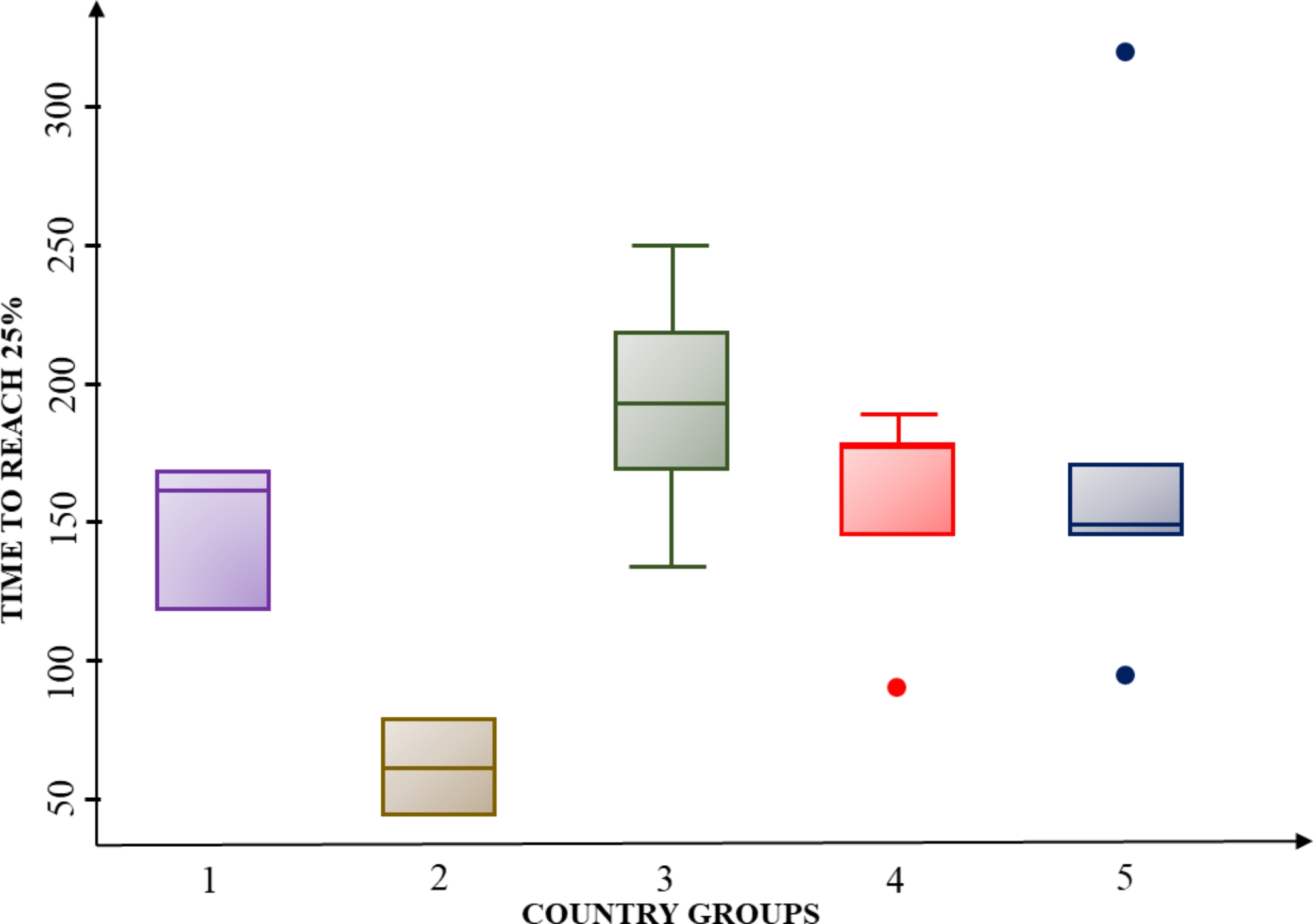
Boxplot for time to reach 25% (TTR25)

**Fig. 5:**
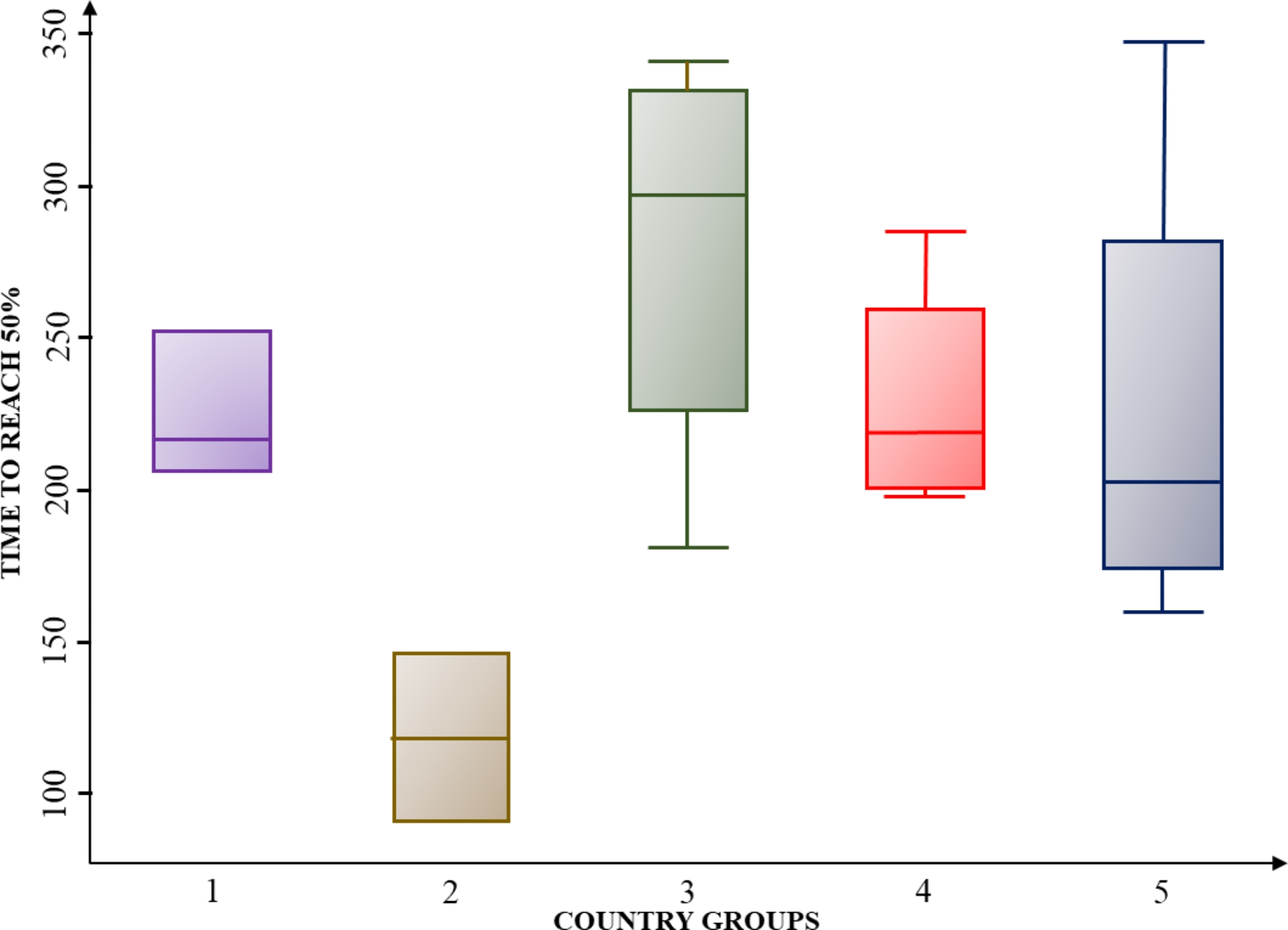
Boxplot for time to reach 50% (TTR50)

**Fig. 6:**
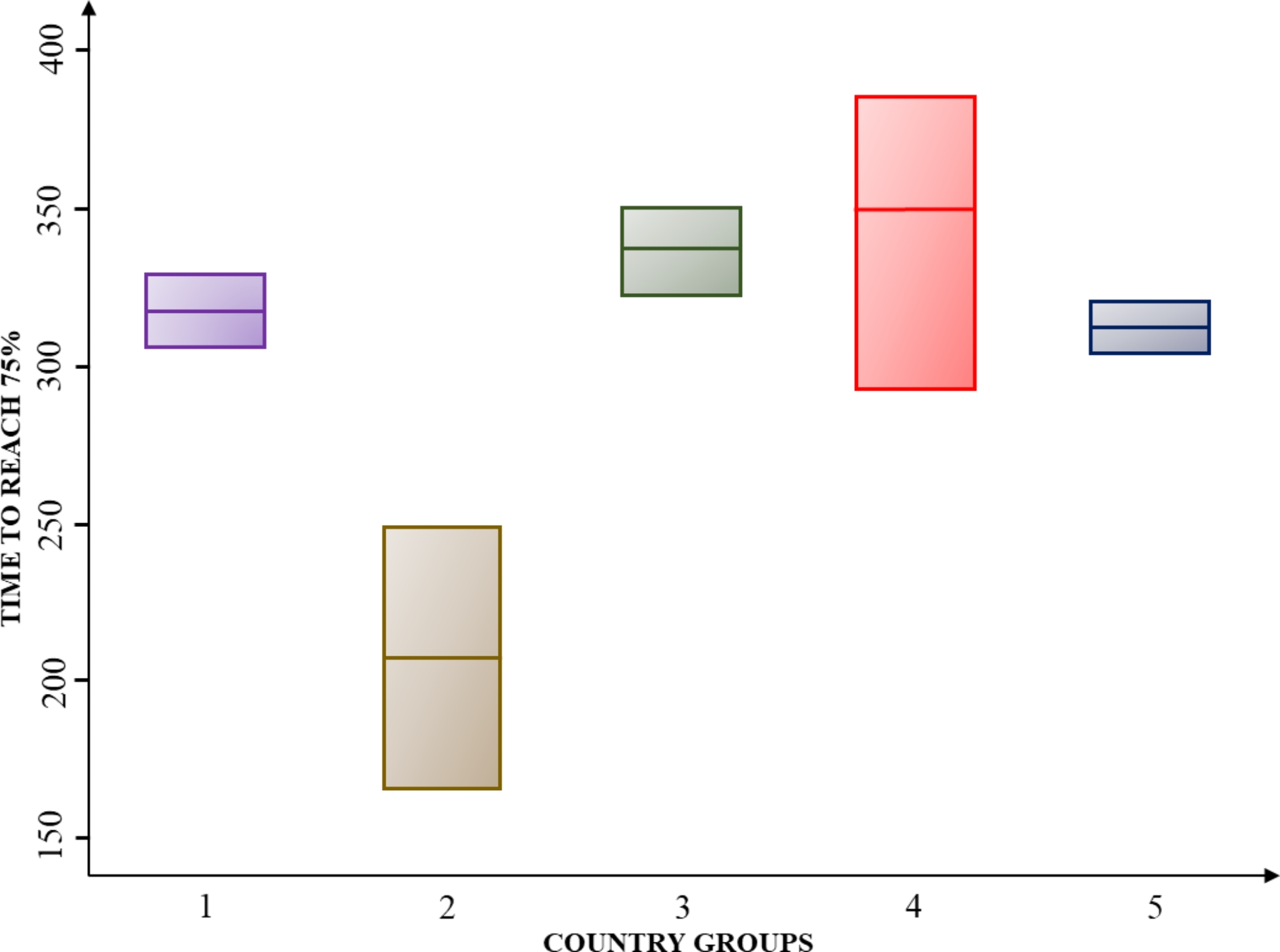
Boxplot for time to reach 75% (TTR75)

In the next step, we perform hypothesis testing for analysis of variance to identify whether there were significant differences among the country groups in terms of covid-19 vaccination outcomes. Using these groups as the independent variable, we analyzed variance. We tested the assumptions for conducting the analysis of variance: normality, homoscedasticity, and independence between groups. The normality assumption was verified by performing the Shapiro-Wilk test, where the null hypotheses of the normal distribution of data were not rejected due to high p-values. The homoscedasticity assumption was tested using Bartlett’s test in Stata and graphing the residuals of the model, both tests suggested homogeneity of variances. The independence of data was ensured in the sampling stage, we carefully selected from SEDA index database the data from each country regarding their overall score for the year 2019, which were independently rated. The results showed significant differences between groups when considering the time to reach 25% (TTR25) and 75% (TTR75) of vaccinated persons, with a p-value lower than 0.1 in both cases. The boxplots (See Figs. 4 and 5, and 6) show that group G2 (Uruguay and Chile) is the only one that has a significantly different performance from the rest of the countries. Graphically, the mean and values of G2 never intersect with those of the other groups since the days to reach the percentages are always shorter for countries in this group. In the TTR25 boxplot (see Fig. 4), the outliers in G5 are Jamaica (too slow for that group and the region in general) and Dominican Republic (too fast for that group). In group 4, the outlier is Suriname (too fast for that group).

In addition, for all the groups the internal variance (box size for each group) is smaller for the TTR25 and TTR75 analysis. The variance in all groups started being small in the 25% cut-off and increased in the 50% cut-off, while the analysis for the independent variable of TTR50, is not statistically significant (p-value equal to 0.11), the boxplot shows how internal variance is higher and the minimum and maximum group values have a significant distance. Subsequently, in the TTR75, the internal variance was lower for G1, G3, and G5, but higher in G2 and G4 it increases.

The above-presented results considered a 0.1 level of significance selected because it represented the level at which these results were valid. We accepted the results as valid at this level by contrasting with the box-plot analysis the variance differences between groups and finding coincidence with the numeric results. Thus, in that sense 0.1 shows the level of confidence on which actors of health systems can count for orienting their decision-making.

### Second phase: qualitative results and analysis

The previous analysis showed that in the case of G2, there was a clear correspondence between the outstanding SEDA index and the outstanding vaccination outcomes, leading to show high resilience. In contrast, for the other groups, intragroup variability prevented us from concluding the relationship between the SEDA index and performance. From there, the sampling for the qualitative phase was derived by considering the countries that represented a success case and the countries that do not show a significant correspondence between the SEDA index and vaccination outcomes to further explore qualitatively the potential intervenient factors in that relationship. On this basis, two groups were selected for the qualitative analysis for a total of seven countries: Chile, Uruguay, G2, and, Colombia, Dominican Republic, Ecuador, Jamaica, and Paraguay, corresponding to G5.

On the one hand, G2 (Chile and Uruguay) was selected because it was the only group that presented significant differences in its displayed resilience concerning the rest in all the ANOVA analyses and internally presented little variance. In this case, it is interesting to understand which part of the high resilience evidenced in G2 is attributable to the vaccination strategy and which to the SEDA index. On the other hand, ANOVA analyses show that there is no clear differentiation between the other groups in terms of the time it takes to reach vaccination coverage, i.e., the SEDA index does not predict resilience in the other groups. In this case, the analysis of the vaccination strategy is necessary to understand which of its dimensions influence resilience. Given that any of the other four groups meet the characteristics sought to study this second aspect, G5 was selected because of the researchers’ interests.

**Fig. 7:**
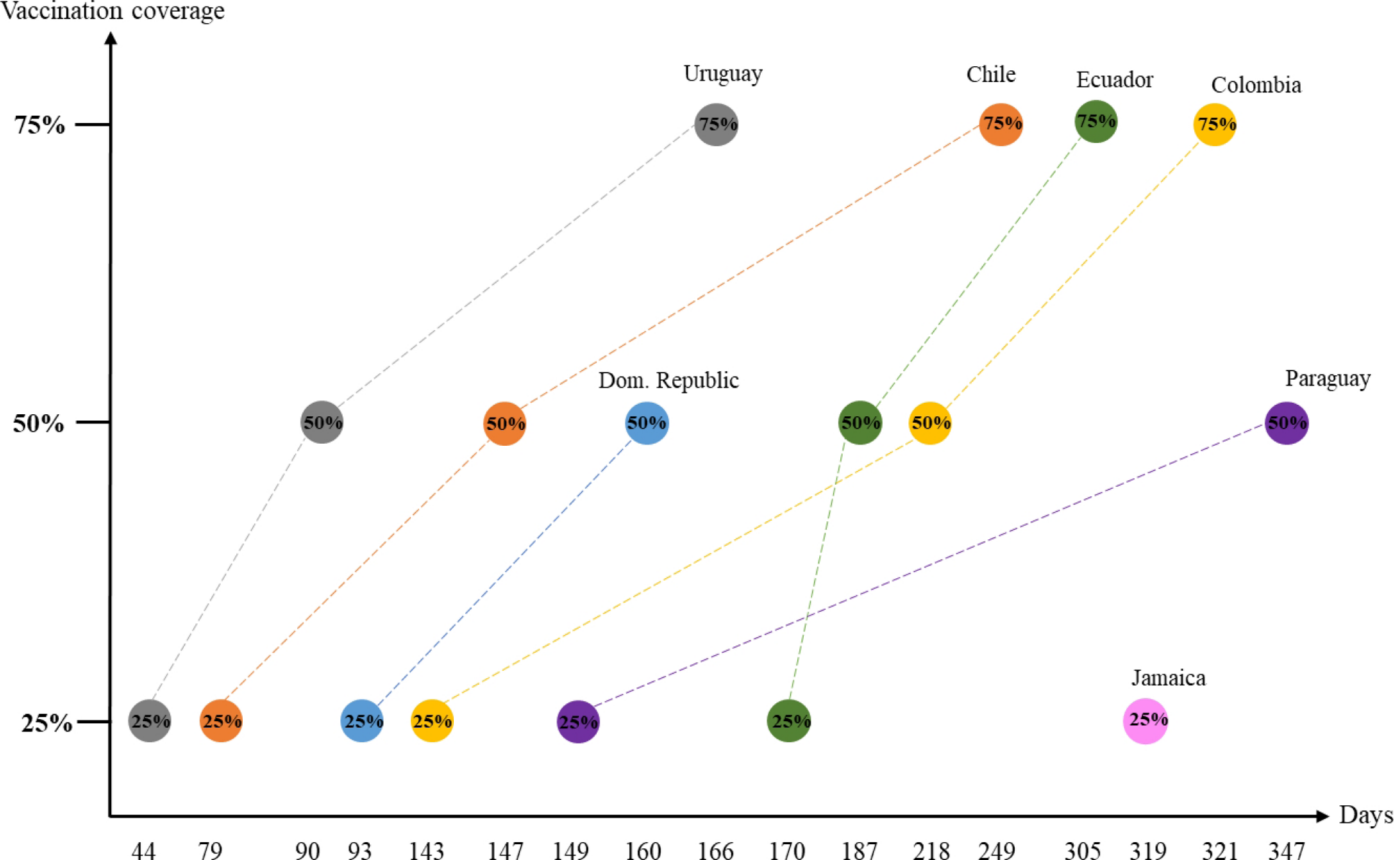
Time to reach 25%, 50%, and 75% covid-19 vaccination coverage

Since this qualitative stage aims to provide an understanding of the reasons for outstanding vaccination performance through pattern identification, the first step was to map the selected countries, outside of the previously formed groups to find new categories based on the vaccination outcomes and later delve into them by exploring their specificities in terms of macro-context background and vaccination strategies. Thus, Fig. 7 shows the selected sample of cases of this second phase and their respective covid-19 vaccination outcomes for the different coverage cut-offs (25%, 50%, 75%) analyzed in the previous phase. Figure 7 shows that four out of seven countries achieved the 75% of covid-19 vaccination coverage (Chile, Uruguay, Ecuador, and Colombia); two countries achieved 50% of coverage (Dominican Republic and Paraguay); and one country achieved 25% of coverage: Jamaica.

Among those that achieved 75% of coverage, Chile and Uruguay (orange and gray lines) had outstanding performance since the beginning of the covid-19 vaccination, and they maintained them throughout the process and completed 75% of coverage in 166 and 249 days, respectively.

Despite Colombia and Ecuador (yellow and green lines) did not get off to such a successful start, in the end, they managed to achieve 75% coverage, not so far behind Chile and Uruguay. The graph shows that Colombia reached 25% of coverage 27 days faster than Ecuador, but then took 75 days to reach 50%, while Ecuador took 48 days. This shows the value of making this measurement through the cut-offs in time because it reflects the learning curves of countries in terms of vaccination, four countries that could be contained in the same category because of their final 75% outcome, can be disaggregated according to their progress throughout time. On the one hand, it can be observed that Ecuador’s learning stage took place between 0% and 25% coverage since the time was longer, and then after 25% was reached, the high slope of its curve reflects that it maintained a very good performance. On the other hand, Colombia’s learning stage took place between 25% and 50% coverage, since the slope is flatter, meaning it took a longer time to achieve that, and then between 50% and 75% coverage, the slope was higher, meaning better performance.

The rest of the countries did not surpass 50% coverage (Dominican Republic and Paraguay) by the time the analysis was conducted, particularly Jamaica did not surpass 25%. On the one hand, it seemed that the Dominican Republic had a good kick-off, since they achieved 25% of coverage in only 14 days more than Uruguay, and their slope was almost the same. However, Dominican Republic did not reach 75%. On the other hand, for Paraguay, it took 149 to reach 25% of coverage and from there, other 198 days to reach 50%, several days in which countries such as Colombia, Ecuador, Chile, and Uruguay have already reached 75% of coverage. Last, Jamaica was the country that took the longest to reach 25% coverage (319 days) and it did not manage to surpass that rate.

The above-presented analysis leaves many ‘whys’ and ‘hows’ unanswered concerning the vaccination outcomes, particularly answering the research question: *How do the covid-19 vaccination strategies affect the HSR?* To achieve that, the remainder of this section presents the results of a documentary analysis on vaccination strategies using a comparative case-oriented approach to report them, taking as a basis for the ‘casing’ process the already identified patterns across countries’ vaccination outcomes. Thus, the countries are grouped into: ‘Early performers’, ‘Prompt performers’, and ‘Delayed performers’.

Table 2 shows a high-level overview of the results of the documentary analysis conducted on vaccination strategies. For this analysis, we used sources such as news, tweets, newspapers, and official government documents, and looked out for the vaccination strategy dimensions mentioned in subsection 3.2: *immunization coverage strategy ratio, vaccination accessibility, and the supply scheme*. It is important to highlight that the scale for each dimension was developed by comparing the characteristics of each country and establishing the thresholds of what could be high or low, considering the internal composition of the sample.

**Table 2:** Covid-19 vaccination strategies

	Country	ICS ratio	Accessibility	Supply scheme
**Early performers**	**Chile**	0.29	HIGH	• Early agreements and negotiations • COVAX: +7 million doses
				• Massive vaccination centers
	**Uruguay**	0.33	HIGH	• Late negotiations • COVAX: 1.5 million doses
				• Massive vaccination centers
**Prompt performers**	**Colombia**	0.42	MEDIUM	• Anticipated negotiations • COVAX: 20 million doses
				• Late opening of massive vaccination centers • Customized vaccination campaigns depending on the population characteristics
	**Ecuador**	0.39	MEDIUM	• COVAX doses: + 3 million doses
				• Massive vaccination centers
**Delayed performers**	**Dominican Republic**	0.38	HIGH	• COVAX doses: +2 million doses
				• Intramural and Extramural vaccination centers
	**Paraguay**	0.38	MEDIUM	• Early negotiation with five pharmaceuticals • COVAX doses: +4 million doses
				• Drive-through and pedestrian vaccination centers • Differentiated locations between weekdays and weekends
	**Jamaica**	0.44	HIGH	• COVAX doses: 124,800 • First country receiving COVAX doses in the Caribbean
				• Mobile and permanent vaccination centers

The remaining section is structured following the cases mentioned in the first column of Table 2. Each case delves deeper into the detail of the vaccination strategies dimensions.

### Early performers

Early performers are those countries that had an outstanding performance since the first assessment conducted at a 25% coverage level of covid-19 vaccination. In this case, Chile and Uruguay were the best countries in the region, taking 79 and 44 days, respectively to achieve that coverage goal. In addition, this performance was sustained over time, and it was also the countries that achieved in the first place the 75% coverage goal.

Chile pioneered the start of the covid-19 vaccination in the region on the 24th of December 2020, while Uruguay started its vaccination roll-out on March 1^st,^ 2021. Before the start of the covid-19 vaccination roll-out, Chile negotiated an agreement between the Catholic University and Sinovac that resulted in preferential access to dose supply once all the regulatory agencies approved its use [33]. In contrast, Uruguay was one of the last countries to enter the vaccine negotiation and procurement phase, which resulted in the late arrival of vaccines. This was because they were waiting for the trials to be more advanced and acquire the highest quality vaccines [34].

The **immunization strategy** of these countries was balanced in terms of increasing population coverage while completing the initiated schemes. This balance manifests in the ICS ratio, which takes a value of 0.29 for Chile and 0.33 for Uruguay, between 0 and 0.5, closer to 0.5, which means a mid-position slightly leaned over inoculating most people with two doses, which is consistent with their initial goals of fully vaccinating 80% and 70% of the population, respectively.

In terms of **supply scheme**, both countries used massive vaccination centers, Uruguay started with 90 vaccination centers, 46 in Montevideo and 45 across the regions; and, in Chile, vaccination centers were in mass venues such as universities and stadiums in all 16 zones of the country, with more than 1400 vaccination centers. Also, the personnel authorized to vaccinate included nurses, nursing technicians, and other health professionals such as midwives and dentists [35].

Regarding **accessibility**, both countries leverage technology and IT systems for communicating and scheduling the population. In Chile, an online calendar was established to guide the population on their eligibility status to access covid-19 vaccination, according to the stages established in the national plan. In Uruguay, the collaboration between private and public organizations such as the Ministry of Public Health of Uruguay and the Information and Knowledge Society (Agesic) made it possible to quickly design an IT tool that operated as a scheduling system through different communication channels based on an app previously designed for monitoring covid-19 in the country. This system managed around 800,000 requests and registrations daily and had the advantage of being synchronized with the government and health providers’ software. This was translated into high accessibility since people could keep a single vaccination record, and their vaccination schedule could be tracked from any institution at any time [36].

Overall, the key mechanisms that allowed Chile and Uruguay to be early performers and sustain that throughout time, were the few restrictions in access given by massive centers, the flexibility on the vaccination schedule provided to the population, the usage of IT systems for communicating and scheduling and their adequate integration with the health service providers and vaccination centers; and the role collaboration and cooperation with international parties and academia, in the case of Chile, and across public organizations in the case of Uruguay.

Before the covid-19 emergency, both countries, Chile and Uruguay, have devoted significant efforts in the last 15 years to orienting their system toward a universal health coverage approach and reducing inequality and market-oriented health services. In Chile, in 2002, there was a reform to the health system that aimed to decentralize it through the creation of the Health Care Network Integration Councils (CIRA), which followed a logic of distributive justice and aimed to better connect the system by addressing service gaps in some regions. However, it is a purely supportive figure, meaning it has no legal implications or power in resource allocation processes. Nevertheless, it is considered an essential first step toward improving governance, particularly accountability for health services [37]. In Uruguay, in 2007 a system reform was carried out focusing on three core goals: change in the financing model, change in the management model, and change in the care model [38]. As a result, there have been some critical milestones in the country since then: the creation of an advisory council for the design of health policies, the formation of the national health board that has legal powers to manage the national health insurance, and the directory for the management of public health services [39].

These cases share reforms aimed to foster governance by including the community, forming new participation mechanisms for decision-making, and redistributing their control and financial means to reduce inequality. This helped them be prepared for the covid-19 emergency with better communication and assessment of the service coverage in the country, which was vital for designing a mass vaccination strategy.

### Prompt performers

Prompt performers are those countries that did not perform outstandingly at the early stages of covid-19 vaccination, i.e., 25% cut-off, but that showed a considerable improvement in the latter stages of vaccination, i.e., between 50% and 75%, suggesting learning over time. In this case, Colombia and Ecuador were the prompt performers, taking 143 and 170 to achieve 25% of vaccination coverage; and further 178 and 135 days to reach 75% coverage, respectively. Ecuador started vaccination in mid-January 2021, and Colombia started the vaccination roll-out on February 17th of 2021.

The **immunization strategy** of both countries was mainly oriented toward completing schemes rather than covering people with at least one dose. This priority manifests in the ICS ratio, which takes a value of 0.39 for Ecuador and 0.42 for Colombia, between 0 and 0.5, closer to 0.5, which means a position predominantly leaned over inoculating most people with two doses. However, both countries started with low vaccination rates and high accessibility barriers, such as long waiting lines and a lack of coordination between healthcare providers and vaccination centers.

In terms of the **supply scheme**, at the beginning in Ecuador, the approach to covid-19 vaccination was marked by a division between public and private institutions [40], which represented a problem and slowed down the vaccination rates. This changed radically with the possession of the new president, and Ecuador went from being a critical case to a reference in the region. The new government integrated the capacities of public and private institutions. It developed joint logistics involving nearly 3,100 private companies, universities, armed forces, police, firefighters, the Red Cross, and local governments [40]. The vaccination centers were in the sites where the last elections had been held; stadiums, coliseums, and mobile brigades that accessed the most remote areas with the help of the air force [41].

In Colombia, the **supply scheme** considered a highly dispersed population and more than 2 million migrant populations [42] and designed customized strategies according to regions. For example, through intersectoral public-private alliances, all Amazonian regions began to be inoculated independently and solutions were created for vulnerable and peripheral populations that could not access urban vaccination centers. In some areas, this materialized in mobile brigades, itinerant vaccination centers, and extramural vaccination campaigns. However, as well as in Ecuador, in the early stages, there were also pitfalls regarding the supply scheme and the capacity of the vaccination centers, which were a consequence of fragmentation between healthcare providers (public and private) that remained operating at their centers, generating a long list of offerings for the population but with limited capacity each. In mid-2021, this condition changed radically, and mass vaccination centers located in stadiums, universities, and shopping malls were opened, thus covering a more significant percentage of the population.

Regarding **accessibility**, in both countries, this started being low as a direct consequence of the lack of integration between public and private schedules, priorities, facilities, and logistics. Thus, the improvement in accessibility was also a direct result of the increase in physical capacity and the joint efforts among actors. However, there were particularities to each country in the early stage of vaccination that also contributed to the changes in accessibility to vaccination.

In Colombia, there was at the beginning an additional barrier to accessing covid-19 vaccination, mainly because the eligibility criteria at mass vaccination centers involved more than simply belonging to the current stage of the national vaccination plan. A mandatory process for assigning and scheduling patients to vaccination centers requires multiple interactions and reports between organizations [43]. This caused several drawbacks in the progress of the covid-19 vaccination roll-out, as the patient needed more flexibility, and there were IT inconsistencies in patient records that led to confusion in the population and delays in the vaccination. This changed in mid-2021 due to the elimination of additional requirements and the integration of public and private schedules of vaccination stages. Thus, the access was a changing condition that started being low and, after six months of operations, became high.

In Ecuador, during 2020, the impact of the pandemic was very intense, reaching excess mortality levels of over 200%, by March 2020, it was one of the territories with the highest infection and death rates in the world, and this situation did not improve throughout the rest of the year, hospitals were overloaded with cases and they experienced difficulties in keeping track of infections, deaths, and their reasons [40]. By the end of 2020, Ecuador had more than 200,000 cases and nearly 14,000 deaths, which generated high rates of fear and horror in the population, this might have influenced the willingness to get vaccinated in the population, since there is evidence that a greater extent of fear to get infected of covid-19, decrease 33.71% of hesitancy towards covid-19 vaccine than those that have not fear at all [44]. However, in 2021 when the vaccination started vaccination centers were overcrowded and the waiting lines were of several hours, thus the government ask private universities and other institutions for support in the process, but the accessibility remained low, until the change of government and the initiatives that were mentioned in the supply scheme section.

Overall, the key lessons from the *prompt performers* are the value of learning and being flexible enough to transform the strategies on the way. However, achieving this implies cooperation among actors and across sectors because of (i) the complex challenges of massive vaccination logistics, such as accessing rural populations, or increasing physical capacity; and (ii) the need for resources and facilities that do not originally belong to the health care providers, such as stadiums and malls. The macro-context background in these countries manifests in the preconditions of the health system such as their fragmentation and the previous IT difficulties, multiplicity, and inconsistencies in patient records, that accentuated the challenges during covid-19 vaccination. Also, the improvement of Ecuador was mainly due to a change of government that led to a change in the logistic approach.

### Delayed performers

Delayed performers are those countries that by the time of the study have not achieved more than 50% of covid-19 vaccination coverage: Dominican Republic, Paraguay, and Jamaica, the latter did not surpass the 25% cut-off. The Dominican Republic had a good kick-off, since they achieved 25% of coverage in only 14 days more than Uruguay, while Paraguay and Jamaica, were achieving the 50% and 25% coverage, respectively, by the time the rest of the countries in the analysis have already reached 75%. The vaccination roll-outs started on the 16th of February 2021, in the Dominican Republic; on the 22nd of February 2021 in Paraguay; and on the 10th of March 2021, in Jamaica.

The **immunization coverage** of the three countries was highly oriented towards completing schemes, Dominican Republic and Paraguay had the same ICS ratio, of 0.38; and Jamaica had an ICS ratio of 0.44. The closer the ratio gets to 0.5, it means a position predominantly leaned over inoculating most people with two doses. However, it is essential to note that the immunization ratios in Jamaica were affected by a low willingness to be vaccinated in the population, which also resulted in more than 40 vaccination centers being empty daily and over 25 thousand people that have passed the 8-week date without receiving the second dose [45]. Regarding the Dominican Republic, Fig. 5 showed that it started well and the progress towards completing 50% was almost as fast as the case of Uruguay, however, the country did not surpass that rate. This can be explained by the fact that there was a significant increase in this ratio due to restrictions imposed by the government to enter shopping malls, public transportation, and other public places in the city that required the complete vaccination scheme. Daniel Rivera, Minister of Public Health, said that since restrictions were announced, the daily rates quadrupled, reaching more than 55,000 people vaccinated in a single day [46].

The **supply scheme** was similar for the three countries since they all have two types of locations: mobile and permanent vaccination centers. The first one was to reach rural populations or specific communities and the second one was located at strategic points in the cities such as health centers or churches. Particularly, the Dominican Republic’s national vaccination plan specified two types of vaccination centers: extramural and intramural. The Intramural ones were established with health facilities that provide specialized care to patients in risk groups and nursing homes. The extramural ones consisted of fixed vaccination centers located in strategic places in the different communities and mobile points to reach remote communities [47]. The main challenges in terms of supply were reaching remote areas of the country with poor infrastructure in the case of Paraguay and negotiating with the anti-vaxxer’s movements in the case of Jamaica.

The **accessibility** was high for Jamaica and Dominican Republic and medium for Paraguay. As mentioned earlier, Paraguay faced important challenges in making available the vaccine for rural populations, which is why the punctuation given on this matter is medium. However, Paraguay as well as the other two countries leverage technology for the scheduling processes, the Paraguayan Ministry of Information and Communication Technologies developed a system for covid-19 vaccination that allowed the population to register themselves and schedule their appointment for vaccination, considering the eligibility criteria according to the national plan and patient availability. The Dominican Republic used digital mechanisms was foreseen to schedule vaccination appointments according to the phases of the national plan. Jamaica designed a website as a solution to be used at the national level for all covid-19 related issues, that allow scheduling appointments for eligible individuals according to the stages of the national plan [48]. In addition, walk-in vaccination centers and call centers were available for people not using digital tools.

Overall, the key lessons from *delayed performers* are that the willingness of the population to get vaccinated is always a high-level priority because, without that, any digital efforts, supply facilities, or immunization strategies do not work. There are two clear examples of this, first, the Dominican Republic implemented incentives for vaccination by imposing restrictions on access to public spaces, which turned out to be a short-term solution to increase the rates, but when the restrictions were dropped, the vaccination rates fell too. Second, Jamaica had multiple vaccination centers that consider the characteristics of the population, and they have high accessibility to appointments, but they were one of the three countries in the Pan American Health Organization region that did not meet the WHO standards of inoculating at least 10% of their population by September 2021 because of the low willingness in the population. The Paraguayan case reinforces the importance of macro-context background since it is one of the countries with the highest proportion of the rural population in the region and the poor conditions in some regions hindered the progress of covid-19 vaccination.

## Discussion

The HSR literature establishes four stages to observe resilience across the shock cycles, which are: preparedness for shocks; shock on set and alert; shock impact and management; and recovery and learning [8]. In this study, preparedness can be observed in the 25% vaccination coverage cut-off, in which Chile and Uruguay took the fewest number of days to reach that quota, and the box-plot analysis showed low variance between them. While for the rest of the countries, there was a higher number of days to reach 25%, but still a relatively small variance inside the country groups. This provides insights about the initial stage of crisis handling in terms of its resilience; by TTR25 analysis, no country knew the correct way to carry out mass vaccination, and all of them were in an experimental stage, figuring out how to deal with this challenge. Moreover, through the early performance, i.e., the 25% coverage vaccination outcome, the ‘early achievers’ show how a macro-context background with previous health system reforms oriented towards a universal health coverage approach; and reducing inequality and market-oriented health services. This allowed Chile and Uruguay to have a better baseline for guaranteeing equal access to vaccines and establishing cooperative and collaborative dynamics among actors, instead of market-oriented relationships. In contrast, Colombia and Ecuador suffered the consequences of not being prepared in terms of ‘effective information systems and flows’ [8]; they had previous IT difficulties, multiplicity, and inconsistencies in patient records, that accentuated the challenges during the covid-19 vaccination. As well as a highly fragmented system that hindered the ‘coordination of activities across governments and key stakeholders’ [8].

Then regarding the stage of shock on set and alert, a timely identification requires ‘robust and comprehensive surveillance and early warning systems’ [8]. This is an opportunity window for Latin American countries and future research because, in the documentary analysis, we did not identify anything related to alert systems that helped to preempt the covid-19 crisis, particularly regarding the vaccination challenge. However, we identified at this stage the value of getting involved early in international negotiations to guarantee access to vaccines. There is an unexplored aspect that could orient future research regarding regional on-set and alert. How could we design regional effective surveillance mechanisms that reduce uncertainty and dependency on developed countries for future crises?

The shock management [10] studied through the comparative analysis of vaccination strategies, showed the complementarities of immunization coverage, accessibility, and supply scheme. The prompt performers are the best example of how changes in logistics, alliances between public-private sectors, and removing access barriers can positively influence vaccination outcomes. The analysis of variance showed that in all groups the internal variance increased between the 25% and the 50% cut-off, which can be explained by the differences in crisis management presented in the qualitative cases, there are multiple combinations of immunization approaches, accessibility, supply scheme and willingness to get vaccinated in the population. However, shock management is subject to the country and population conditions. The main challenges in achieving vaccination outcomes occurred in Paraguay, due to the high amount of rural population and the difficulties in reaching it; the low willingness to get vaccinated in Jamaica despite de high accessibility; and the short-term incentives that did not motivate the population properly and could not surpass the 50% coverage rate.

Last, the learning and transforming from shocks which were approached through the study of vaccination outcomes across the 25%, 50%, and 75% coverage cut-offs, can be seen as the value of this type of assessment since it allows to recognize the changes of patterns along the curve and identify the stages of learning across the whole vaccination roll-out. The changes in the slope of the curves that showed improvement in efficiency throughout time are a clear reflection of health system learning. In the TTR75 cut-off, the internal variance was lower for G1, G3, and G5, which could be explained by the learning and replication of practices that prove to be effective in other countries of the region, such as massive vaccination centers or removal of access barriers that countries such as Ecuador and Colombia adopted later than the rest show the learning potential that is present when there is an effort to observe the others in the region and adapt accordingly. While, for G2 and G4 the variance in the TTR75 cut-off was higher, which can be explained by the changes in the immunization approach and strong contextual differences between countries, despite the strategies in vaccination impede a homogeneous performance.

## Conclusion

The main research problem that guided this work was the search for a complex, multilevel perspective on HSR during the covid-19 emergency. The mixed methods design allowed us to show that exceptional levels of societal well-being translate into high and sustained levels of performance in covid-19 vaccination, thus, high resilience. Whereas, for countries with average and lower scores in the SEDA index, the operational decisions and the learning on the move regarding their own and their peers’ trajectories were crucial and were reflected in the changes in covid-19 vaccination outcomes for the different coverage cut-offs (25%, 50%, 75%) analyzed. This study contributes with its analysis approach of vaccination strategies using cut-off points that allow a performance view that takes into consideration the stages of the vaccination progress and the learning process that goes with it, and this approach can be transferred to assess regular vaccination programs as well as a framing of the covid-19 vaccination experience into the HSR shock cycles, which provides an opportunity for further research to explore a longitudinal view of HSR complementing the cut-offs and the stages of cycles.

## Data Availability

The datasets supporting the conclusions of this article are available in. OWID: https://ourworldindata.org/covid-vaccinations. SEDA index: https://www.bcg.com/industries/public-sector/sustainable-economic-development-assessment.

## Footnotes

1 Boston Consulting Group (BCG) is a global consulting firm founded in 1963. Today, they partner with leaders in business and society to tackle their challenges and capture their greatest opportunities.

## Abbreviations

ANOVA: Analysis of Variance
HSR: Health System Resilience
ICS: Immunization Coverage Strategy
SEDA: Sustainable Economic Development Assessment
TTR: Time to Reach
